# Between reluctance and resistance: LGBTQ+ healthcare professionals and their experiences in the workplace

**DOI:** 10.3389/fpubh.2026.1903749

**Published:** 2026-08-11

**Authors:** Mara Pieri

**Affiliations:** Centre for Social Studies, University of Coimbra, Coimbra, Portugal

**Keywords:** coming out, diversity, health professions, healthcare, LGBTQ+

## Abstract

**Background:**

Literature shows that stigma against LGBTQ+ people in healthcare affects professionals as well as patients. Research indicates that discrimination is transversal to all healthcare professions; hierarchies and heteronormativity contribute to difficult visibility both with colleagues and with patients.

**Objective:**

This study aimed to explore the experiences of LGBTQ+ healthcare professionals in the workplace, analyzing their practices of (in)visibility, their experiences of discrimination and their relationships with patients.

**Methods:**

Data were collected through semi-structured interviews to 17 healthcare professionals working in the public healthcare sector in Portugal and identifying as LGBTQ+. Data were analyzed through thematic analysis.

**Results:**

Healthcare professionals report challenges in managing visibility in the workplace and the relevance of verbal abuse in interprofessional relationships. They oscillate between reluctance to assume their identity publicly and micro-practices of resistance to discrimination.

**Limitations and future directions:**

The findings of the study stress the importance of micro-practices in the definition of LGBTQ+-friendly healthcare environment and the need for further research on visibility in the healthcare workplace.

## Introduction

Studies on LGBTQ+ experiences in healthcare show that stigma against LGBTQ+ people is present in healthcare contexts as in all the other sectors of society (1). However, the literature mostly focuses on the experiences of LGBTQ+ patients and less contributions are dedicated to the experiences of LGBTQ+ healthcare providers (HPs) (2, 3).

Studies reveal that the stigma against LGBTQ+ professionals is transversal to all healthcare professions: for example, radiation therapists (4, 5), pediatricians (6) and nurses (7–9). Relevant research analyses the experiences of LGBTQ+ students in medicine (10, 11) and trainees (12), showing similar results. This corpus of literature highlights that regardless of their specific role, HPs generally face stigma, discrimination, and additional obstacles in their daily work. Some studies also point out that HPs who are racialized, have disabilities, are chronically ill and/or have low-class backgrounds are more exposed to multiple forms of discrimination (13). Negative reactions by co-workers and patients, as well as fear regarding their physical integrity, result in higher risks of discrimination and poor mental health (14, 15). Patients are often agents of discrimination themselves and reproduce the homo-transphobic cultural milieu of society in general (16).

In light of the need for healthcare workplaces to be more inclusive and welcoming for LGBTQ+ people, some institutions adopted non-discrimination policies in the past decades, especially in the North American context (17). Moreover, curricular units dedicated to LGBTQ+ issues during medical school and training became more common (18, 19). However, most of them are directed at improving the treatment of LGBTQ+ patients while maintaining silence on the experiences of LGBTQ+ healthcare workers. This gap contributes to reinforcing a spiral of silence (20) on LGBTQ+ issues in healthcare and to reproducing the assumption that all HPs are cis/heterosexual.

Healthcare environments are particularly characterized by hierarchies in power relations (21, 22) and heteronormative dynamics (1, 23). Before coming out, HPs evaluate a vast array of issues, such as the lack of knowledge about LGBTQ+ issues, the religious or political ideals of co-workers, and the risk of suffering harassment (24). Coming out is considered potentially disruptive also because it is perceived as a threat to the professionality and reputation of HPs (5). However, HPs recognize that identity disclosure could benefit their working environment and provide an important contribution to making LGBTQ+ people, including patients, feel more welcomed (4). Parker-Der Boghossian, in this regard, notes that: “it is one thing to serve LGBTQ patients with culturally responsive programming but it is quite another thing to recruit, hire, and retain LGBTQ staff at all levels of the organization; especially if the LGBQ staff are empowered to assess and contribute to the work of LGBTQ health equity” (25) (p. 126).

Studies focused on well-being and performance at work show that the pressures experienced by LGBTQ+ healthcare providers may lead to worse health outcomes in later life, substance abuse, mental health issues and burnout (7, 26, 27). The pressure to (in)visibility often leads to hypervigilance, stress, and anxiety (28, 15). Finally, the pandemic of Covid-19 intensified working precariousness and harshened pre-existing factors of stress (29). In recent decades, healthcare systems in Europe have been encouraging Diversity, Equality, and Inclusion (DEI) policies. Measures to encourage the employment of immigrant HPs, establish culturally oriented care, and promote a culture of respect have contributed to improved diversity scores within the healthcare profession (30, 31). However, DEI programs have been increasingly criticized under the growth of far-right groups and anti-scientific movements (32). Attacks to DEI programs have targeted especially LGBTQ+ people and immigrants, fostering hostile narratives and resisting recognition of the structural inequalities—such as racism, homo-transphobia, and sexism—that continue to affect healthcare professions (1). In this context, HPs who also identify as LGBTQ+ nowadays seem to face multiple struggles that may alter their role as potential actors in the advancement of equality and inclusion in healthcare.

In Portugal, literature on LGBTQ+ healthcare investigates in particular the perspectives and experiences of LGBTQ+ patients (33–36). Obstacles to visibility are still predominant and episodes of discrimination are common in the Portuguese healthcare system (37–39). It is worth noting that, in addition to the rapid and multiple changes that occurred in the recognition of LGBTQ+ rights in the past two decades (40), in 2019 the Ministry for Health launched the first National Strategy for LGBT health: the memorandum contains guidelines to improve policies regarding LGBT people in health and was met with great hope by the LGBTQ+ communities. However, the National Strategy does not specifically mention LGBTQ+ healthcare workers as beneficiaries of the policies. The national healthcare system (SNS) offers free, accessible and equitable healthcare to all people living in Portugal, although in the past decades pressures on the costs and an increasing crisis in the model have led to the growth of importance of the private healthcare sector. Finally, since 2021, far-right groups, mainly converging toward the political party Chega, have gained increased traction, especially with an anti-immigration and anti-diversity discourse that seem to hinder the advancements in LGBTQ+ rights happened since 2000 (41).

## Methodology

### Data collection

The data discussed in this article were collected during the second phase of an 18-months research project on the experiences of LGBTQ+ healthcare professionals in Portugal. The study was approved by the Ethics Committee of the Centre for Social Studies of the University of Coimbra. During the first phase, an online survey was disseminated and open for any healthcare professional in any category or role that identified as LGBTQ+ and worked in the country. At the end of the survey, participants could enlist to be interviewed in a second round, involving semi-structured interviews. The interviews were conducted mostly online; the audio was recorded and the content transcribed and anonymized. All interviews were associated with a pseudonym to protect the interviewees’ privacy. They were all conducted in Portuguese and translated into English afterwards for publication purposes. Interviews had a semi-structured script, with a set of open questions on three main topics: (a) relationships with colleagues; (b) relationships with patients; (c) future trajectories and hopes.

### Participants

Seventeen interviews were carried out to healthcare professionals who self-identify as LGBTQ+ and worked in Portugal at the time of the interview. Most of them (*n* = 10) are between 25 and 34 years old; five are between 35 and 44 years old and two are between 45 and 54 years old. As for what concerns sexual orientation, nine interviewees identify as lesbian, six as gay, two as bisexual and one as pansexual. Most participants identify as cisgender (*n* = 4 men, *n* = 11 woman), two as non-binary and one as trans. The geographical distribution is quite even, with the urban areas of Porto and Lisbon more represented than other areas. In terms of professional role, nine are medical doctors (with a specialization), three are resident doctors, and five are nurses. All interviewees primarily work in the public healthcare system; some medical doctors also perform private consultations as a second job.

### Data analysis

Data collected were analyzed through thematic analysis in MaxQda. The thematic analysis followed the six-steps recommended in Braun and Clarke (42), where an initial familiarization with data leads to the identification of recurrent themes and emerging codes. In the first phase, the author identified relevant quotes that showed interesting patterns and vivid depictions of the lived experiences (43). The selected quotes were grouped together under one or more keywords; keywords were then grouped into codes generated during the process. A final set of 19 codes were created to guide the analysis of data. The codes were then processed into five main themes: (i) (in)visibility in the workplace; (ii) experiences of discrimination; (iii) power dynamics and imbalances; (iv) temporalities: changes during time and visions of the future; (v) best practices and recommendations. For the purposes of this publication, only the excerpts assigned to the first three themes were considered.

## Results

Based on the research information and the analysis process, three main themes were identified: (1) experiences of discrimination; (2) practices of (in)visibility; (3) relationship with patients.

### Theme 1: experiences of discrimination

In line with similar studies conducted in the past (5, 8, 13), the results of the interviews suggest that LGBTQ+ healthcare professionals experience and witness a wide range of discriminatory practices in the workplace. Direct discrimination takes often the form of derogatory comments and aggressive behaviors from colleagues, especially when hierarchical disparities are at play. In some cases, interviewees report having suffered mobbing practices:

*For a year they suspected we had a romantic relationship when we only had a friendship. And from that point on, when we actually did start a relationship, it went from being forbidden to work together, having our work called into question, having our personal belongings vandalized, to a negative performance review. […] We went through a rough patch, we had to resort to legal means. (Maria, nurse)*

Performance reviews, which are common for HPs at certain stages of their career, are mentioned as typical moments when line managers operate discrimination by undermining the HP’s work on account of their sexual orientation or by providing unfair assessments of their skills. The consequences of these practice may prove quite relevant for some HPs, especially at the beginning of their career:

*There was another situation, with at least two people I can remember, who were treated more aggressively, it almost seemed like there was always a grudge, always something to pick on. […] One of them was there doing his training, he ended up dropping out of the placement altogether, had to go on psychiatric leave and never came back. (Joana, medical doctor)*

Other interviewees mention also outing as a common practice they either directly experienced or witnessed in the workplace: often it is a revelation of someone’s gender identity or sexual orientation accompanied by a derogatory comment. This seems to be the case especially for those workers who do not conform to gender expressions or have an appearance that defies the normative gender binaries.

However, the most commonly reported forms of discrimination are verbal abuses, such as insults, negative jokes or offensive slurs. These seem to circulate in particular during informal moments, such as smoking or coffee breaks. While in the situations in which patients are present HPs seem to uphold to minimum standards of respect, in these more relaxed instances it is quite common for language and manners to be less controlled, as the following examples show:

*There was this other specialist […] basically, it was her talking about another nurse whose husband had divorced her and was now with a man. And that’s when the comment came up about how all gay men want to first be with a woman, she even said to ‘plant their seed.’ And I was just sitting there like, ‘I can’t believe this is actually happening.’ (Matilde, medical doctor)*

*I had this one time a heated argument with a colleague, without sharing my personal life, though. […] A lot of it kicked off because of Ronaldo’s son …had to be carried by a surrogate. And then that conversation moved on to… same-sex parent families. And this colleague of mine, who's pretty conservative on these things, started criticizing it. And that was really getting to me. Probably also because I’m homosexual, and feeling that I’m capable—I mean, I have two daughters—of being a good mother. (Laura, medical doctor)*

The reaction to these comments or conversation is not always openly confrontational: while some may decide to take the opportunity to react to homophobic comments, others simply silently note the fact without taking any action. In Laura’s experience, it is indeed interesting to note that she reacted to homophobic comments by her colleagues engaging in a “heated argument,” without sharing any element of her personal life, hence not coming out as a lesbian mother of two kids.

The behaviors registered during direct interaction do not always correspond to what happens behind someone’s back, contributing to a culture of fear and mistrust:

*There was a lot of murmuring going on. But when they spoke to us directly, they were supportive. So, I learned that, in fact, I cannot rely on others. I have to rely on myself, I have to build within myself mechanisms that allow me to actually feel grounded and secure without being afraid that others might throw me off balance. (Eduarda, nurse)*

Some interviewees also report that casual homo-transphobic slurs are commonly shared during everyday working activities, to comment daily news or to intervene in a general conversation. Although they generally recognize the harm such comments may generate, interviewees usually do not react, as they intend they are part of a general heteronormative culture in which homo-transphobia is casually reproduced without necessarily a specific aim. One interviewee defines them as “locker room talk”; another, points to them as result of lack of awareness:

*All sorts of comments, especially transphobic, are dropped, and so on and so forth, and sometimes I believe they’re not meant in a bad way—I want to believe that, they’re not meant in a bad way—but they come from a place of genuine unawareness of people’s experiences, of what trans people go through, trans people or people from the LGBTQI+ community. (Filipe, nurse)*

HPs affirm they resort to formal complaint or they pursue legal action against discriminatory practices only when the discrimination is blatant, persistent, and overtly demonstrable. In all other cases, they mostly try to talk with the people involved or ignore the episodes.

### Theme 2: practices of (in)visibility

Coming out in the workplace is a processual learning that can take many different forms and change over time (44, 45). Literature often focuses on the obstacles to coming out as one of the main litmus tests to the sense of safety HPs feel at work, what Beagan defines as “the dilemma of disclosure” (28). The interviews show that the choices about visibility in the workplace are generally difficult and only rarely are done without careful consideration of the potential (negative) consequences.

No interviewee declared to be completely in the closet with all colleagues. Most participants came out with at least some colleagues, while a smaller group is visible with all their colleagues and managers. Hierarchical relationships within the workplace and the precariousness that characterizes especially young HPs are two of the most recurrent factors that influence the choices of coming out. Young HPs, such as resident doctors or nurses at the beginning of the career, seem to be more fearful that coming out as LGBTQ+ may impact their career and their position in the system:

*I’m also very young, at the beginning of my career, so that would also make me stand out, and with some fear that it could be perceived negatively, I prefer to keep it to myself; unless it becomes necessary to talk about it, I just keep to my own corner. (Victoria, medical doctor)*

*When I became a specialist, of course, I gained a certain degree of autonomy within the workplace. Because, as a resident, you are always dependent on those who supervise and train you, and if they start excluding you, things become complicated. There’s a kind of game you have to play. (Xavier, medical doctor)*

Progress in the career provides a sense of safety and tranquility that many recognize has been the most important element: when asked about retrospectively thinking about what most changed in their practices of visibility over time, most interviewees reported this as the main difference occurred.

However, HPs are strongly aware that their workplace is marked by power hierarchies and thus learn to recognize where and with whom they can be open, mostly avoiding conflicts with managers and feeling at ease with those who are at their same hierarchical level:

*There is a hierarchical position because I am a resident and the level above is specialists. […] There are people with whom I feel that over time it becomes more comfortable for me to share this, and there are people with whom I have ongoing contact and with whom I already feel I can approach this topic in a more natural way. […] I just think it is more about still adjusting to these people and, I suppose, testing the waters a bit. (Alberto, resident doctor)*

Hierarchical differences are also connected to generational gaps. In a country where LGBTQ+ rights developed rapidly and recently, older generational cohorts are often perceived as less open and more conservative. A common assumption is thus that those who are biographically older will also be less open to diversity and possibly homo-transphobic.

For some participants, changes in their personal life were a pushing factor to come out to colleagues: for example, buying a house with their partner or having a child would force conversations that legitimize coming out in a way perceived as “natural.” Establishing significant bonds with few colleagues and developing friendships is also regarded as an important step to feel at ease in coming out:

*It’s not something I hide, and it’s not something I keep as a secret, or anything like that. There are a few people I’ve told, because I felt that my closeness to them justified it. But not because of the working relationship—that is, I have never come out in a professional context. They are people with whom I developed a relationship, a friendship. (Tomas, resident doctor)*

In the cases in which interviewees showed practices of visibility to most or all colleagues, it is recurrent the reference to the importance of coming out with naturality, or without giving it too much thought: in other words, to use trivial moments in everyday conversations to communicate one’s sexual orientation seems often better than doing it with a lot of preparation and thought:

*All the people I work with—administrative staff, security guards, the doctors I work with — all of them know my sexual orientation. And I make a point of mentioning it in a very casual way, in the middle of a conversation, so that people also understand that it is something completely normal and take it as such. So, I don’t make a big deal out of it or treat it as something secret, because I think the more secrecy people create around it, the more it feeds gossip. So, in that sense, it is quite straightforward. (Eduarda, nurse)*

However, this approach comes with the awareness that the information will travel and be shared with many other people: as highlighted in the previous section, although experiences of direct discrimination seem to be less present than those of indirect one, interviewees are very aware of the fact that comments behind one’s back, rumors, and jokes are everywhere. Interestingly, humor is reported itself as a way to fight back possible discrimination and somehow protect from abuse:

*Everyone knows. Because I do something quite interesting that has helped me […] I often use humor about things, and I say exactly what it is, and by not explicitly naming it I let people draw their own conclusions, because I speak very openly. So, everyone knows. (Sara, nurse)*

When asked about whether they know other LGBTQ+ colleagues in their workplace, most participants affirm they know few. Only in a couple of cases, the interviewee stated they did not know any other LGBTQ+ person working with them. However, in general, HPs rarely discuss issues connected to discrimination, microaggressions or homo-transphobia with their LGBTQ+ peers:

*We talk… a bit about all kinds of topics, mostly casual conversations related to our families. We don’t talk specifically about difficulties within our service. I think that, all things considered, compared to others, we are quite lucky. (Joana, medical doctor)*

*Out of everyone I work with—from the entire clinical staff—I could probably identify only three people who are gay or lesbian, as far as I know. But it operates within a kind of ‘don’t ask, don’t tell’ policy—that’s more or less how it is. People end up suspecting things, but they don’t ask questions. (Teresa, nurse)*

Finally, some interviewees reported that it is common, before deciding where to apply for a job, to consult with other LGBTQ+ colleagues and choose an hospital where nobody reported any incident with discrimination:

*I had a trainee here and one of the disadvantages for her to choose to stay here was that it is a very conservative environment, that people from the LGBTQIA+ community are not really welcomed. It is something we take into account, even when we are choosing our specialty. (Matilde, medical doctor)*

### Theme 3: relationships with patients

Interviews explored several aspects regarding the relationship with patients. Three topics emerged from the analysis as particularly relevant: the choices of (in)visibility with patients; the experiences of discrimination against and from LGBTQ+ patients; and the empathy in the clinical relationship.

Interviews showed that HPs tend to come out to patients less and with less confidence than they do with their colleagues. Invisibility as LGBTQ+ is the most common option and it is generally upheld as an important value to preserve the clinical relationship as neutral as possible. Several interviewees, for example, highlight how coming out to patients hinders the ethical commitment to care for every person in the same way:

*What I feel is that, as a professional, no aspect of my personal life should be shared. […] When a mother, for example, says she’s really worried because her child has a fever, and the professional on the other end says, “Oh yeah, my kids have had fevers too,” I don’t think that’s the right approach. And from my perspective, this applies both to a fever and to being LGBT—I don’t feel the need to disclose that I’m part of the community. I’m supposed to be able to create a safe-space environment without having to say that I belong to it. (Sara, nurse)*

In some cases, invisibility is a conscious choice elaborated after careful consideration of the pros and cons and stems from a personal perspective that still recognizes the possible positive advantages in coming out:

*The way I operate, I think it’s preferable not to bring up the topic and not to come out—which doesn’t mean that sometimes, in certain situations, I don’t feel like doing it. […] I also understand that there’s a perspective that argues it’s important for LGBT people to know who the LGBT doctors are, and that it helps them feel more understood and respected. […] My personal stance and the way I practice is that I don’t do it, […] so that our therapeutic relationship can stay as “clean” as possible, so to speak. (Laura, medical doctor)*

HPs also acknowledge that coming out to patients, in particular to LGBTQ+ patients, may create the feeling that the responsibility of creating safer healthcare environments is limited to the actions of LGBTQ+ providers:

*I think that, more than the professional talking about their personal life, it really has to be a… it has to be a collective effort. The community, allies, everyone. In other words, there shouldn’t have to be this break between personal identity and professional identity. It doesn’t make sense. (Joana, medical doctor)*

A minority of interviewees report coming out when they realize they are dealing with LGBTQ+ patients: this is the case, in particular, of healthcare professionals working with teenagers or young patients, that may feel worried regarding their identity or experience rejection in the family context. In these cases, coming out as LGBTQ+ as healthcare providers is used as a way to show empathy and to provide a positive example of success and resilience.

In some cases, invisibility is a result of heteronormative assumptions automatically operated by patients, especially when it comes to women:

*We end up building some relationships with patients, who always ask things like, ‘Are you married yet?’, ‘Do you have a husband?’, questions about children and so on. But I don’t take it badly and it’s not offensive; it’s simply that basic normative assumption people make when they ask out of friendliness, not out of malice. Still, it immediately assumes heterosexuality in relation to the other person. (Teresa, nurse)*

*With me, people always assume that I like men. No one has ever assumed, in the first instance, that I like women. So, I don’t feel—unless I communicate it—that a patient or a colleague would ever think that about me. (Matilde, medical doctor)*

The experiences of coming out reported in the interviewees, although rare, are generally positive and do not describe backlash or discrimination from patients. Contrary to other experiences emerged in literature (27, 46), no interviewee mentioned the choice of invisibility as a measure to protect their own safety.

When directly questioned about whether they feel their working environment is generally safe for LGBTQ+ patients, most interviewees show great trust in the ability of their colleagues to adopt non-discriminatory practices regardless of their patients’ identities or their own beliefs. When entering a medical profession, HPs promise respect of the values contained in the modern Hippocratic Oath, among which the sentence: “I WILL NOT PERMIT considerations of age, disease or disability, creed, ethnic origin, gender, nationality, political affiliation, race, sexual orientation, social standing or any other factor to intervene between my duty and my patient” (47). The commitment to non-discrimination seems to be highly regarded by all interviewees, in particular to what concerns the relationship with their patients. Experiences of direct, open, and blatant discrimination toward patients are rare. However, most recognize that more subtle, indirect forms of abuse are more common: in particular, the ones exercised through verbal acts, such as derogatory comments behind patients’ back, ridicule nicknames to identify LGBTQ+ patients and jokes shared in spaces where only HPs have access:

*People have, for example, used derogatory terms. […] They’ve used the word “dyke,” for example. I found it a bit unpleasant, […] unnecessary, but I think it really is a lack of awareness—they don’t realize that term can be somewhat offensive to some people. (Victoria, medical doctor)*

*For example, in the hallway outside a patient’s room, referring to a trans man as, “Oh, that transvestite, I’m not going in there.” For instance, the other day a doctor said to me, “I’ll give you 200 euros if you ask that 80-year-old patient what pronouns he identifies with.” It’s hallway humor […] we have a lot of hallway jokes, yes. […] They happen behind closed doors, so to speak, among professionals. (Maria, nurse)*

Aside from what is considered “hallway humor” or comments behind closed doors, more direct forms of discrimination witnessed are not only extremely rare but are generally considered intolerable:

*When it comes to patients, that’s something I would never tolerate happening in front of me. As for patients, I’ve never seen, never witnessed, and would never tolerate any kind of different treatment because of that. […] Even those colleagues whom I know are more conservative because of their religion and may view it more negatively—I know they would never treat a patient differently because of it. (Tomas, resident doctor)*

As for discrimination suffered by patients and/or families of patients, not many interviewees speak about experiences of workplace violence in this sense. The most relevant cases report to situations in which the HPs are confronted with someone’s homo-transphobic views regardless of their level of visibility with patients. This situation may be increasingly common in particular as the share of far-right voters in the country has been steadily increasing in the past years (41). The following example is emblematic of this clash and the inner conflicts it arises:

*I’ve had situations where I walked into a patient’s room and they had extremist propaganda playing on their phone and said, “What if all these faggots were sick,” and I was fully, perfectly aware that the comment was directed at me. And I still had to sit down with the patient and provide care in exactly the same way I do with everyone else. (Filipe, nurse)*

HPs working with aging patients and patients that misuse substances also report incidents where they were confronted with aggressive slurs or offenses stemming from states of alteration or dementia.

Although the non-discrimination vow is a fundamental guiding principle in the clinical relationship, when directly questioned, most interviewees acknowledge they have a special attention to LGBTQ+ patients. Even when they are not out or do not speak about the issue, when they understand they are treating an LGBTQ+ patient some recognize they put an extra-effort in making sure they have an overall positive experience in healthcare. This is also seen as a way of compensating for discrimination or unfair treatment patients may have gone through in their life:

*I probably, unconsciously, try to be more empathetic with that person. If I somehow identify that they are part of the LGBT community, I try to be more empathetic in order to compensate for less positive experiences they may have had. (Teresa, nurse)*

*Maybe I can be more attentive… I feel like I am a little bit, perhaps, more protective of these people and try to understand whether they are being treated well, because I know they may be subject to discrimination out there. (Lara, medical doctor)*

Empathy and compassion are often mentioned as important tools to create a safe and accessible healthcare environment for LGBTQ+ patients. Some HPs recognize that being a part of the community themselves they may be more aware of the struggles and the obstacles experienced by their patients and thus develop an extra-layer of empathy toward them in the clinical relationship, even when they are not out:

*When I have an AIDS patient with a severe infection that brings them into intensive care and is therefore life-threatening, I feel a very deep sense of compassion that I think other people are not able to feel. Because I don’t think people really understand […] what it meant to be gay 40 years ago. And the need there was to hide it, the inability to have a normal relationship, how that led to the only way of experiencing love being through promiscuous contact with other people, and how that may have contributed to the origin of that disease and the fact that it is present there because of that. (Tomas, resident doctor)*

The empathy and the attention stemming from acknowledging similar experiences and belonging is not visible nor declared: it is shown through small acts of care that patients may not even notice. Language is an important instrument to enact such attentiveness:

*The way I do it has to be discreet, because I’m not going to tell people about my personal life either. There are different ways to address these topics; it often involves asking key questions like, “How would you like to be addressed?” There are those hints that we pick up on, and if we live within the community, we’re able to identify and relate to others a bit better. (Filipe, nurse)*

Some interviewees thus acknowledge that even the acts of kindness or additional care are carried out in a general setting where HPs maintain their invisibility and LGBTQ+ issues are discussed only rarely.

## Discussion

### (In)visibility in the ward

The data collected offer the opportunity to elaborate a multifaceted analysis of the experiences of LGBTQ+ healthcare professionals regarding both their workplace practices and their relationships with patients. As literature on the topic confirms (27, 28), the issue of (in)visibility as LGBTQ+ is the most crucial and presents multiple interesting analytical directions. In line with some of the previous literature produced on how HPs come out at work (4, 16), this study confirms that the choice of visibility is not easy nor unthought. However, the interviews demonstrate that HPs take differentiated trajectories of visibility whether there are with their colleagues or with patients.

In the context of workplace relationships, the choices of (in)visibility are strongly informed by the level of power that HPs perceive they have within the hierarchical organization of careers (22). At the beginning of their career, when they are working as interns or resident and they are still subjected to the supervision and evaluation of colleagues from upper scales of career, most HPs perceive that coming out as LGBTQ+ may be a negative choice. The fear is that being known as LGBTQ+ may lead to unfair assessments, obstacles in the career advancement, discriminatory practices or even mobbing and aggressions. In this phase of career, coming out is somehow put on hold until reaching more stable position and younger HPs tend to prefer to give priority to their career security. Some may decide to come out only to few selected colleagues, usually in a similar career level, or to refrain from coming out at all. On the contrary, when they reach a higher position, which also coincides with more power and more security, most declare a shift in the way they consider their identity and do not feel their coming out should hinder their position anymore. This means also that in many cases the people that are in the position of power are also the oldest, not only in terms of career advancement but also biographically: therefore, the generational gap within the workplace may coincide at the same time with a visibility gap and a hierarchical gap.

It is also relevant to note that the reluctance to come out is mostly connected to the fear that visibility may lead to harmful discrimination or workplace violence. This fear seems to stem particularly from the awareness that, while on the surface workplaces may seem open and welcoming, on a hidden level they are characterized by indirect discrimination, rumors, slurs and acts behind one’s back. Henceforth, even when HPs did not directly experience any discrimination directed at them, they are aware this does not equal safety and enact preventative strategies to protect themselves.

The choices of visibility with patients follow different trajectories and are connected to different motives. The expectation that HPs should be as objective and neutral as possible in their clinical relationships is at the basis of every interaction with patients: this value keeps a strong relevance for all interviewees encountered and is the most relevant reasons they mention for not sharing personal details during clinical activity. However, HPs are aware that healthcare environments are founded on heteronormative assumptions (1, 23) and that, in this sense, LGBTQ+ patients may be subject to the same discriminatory treatment they receive: that is, a set of indirect actions about which they may not even be aware. Despite growing literature on workplace violence regarding attacks on HPs from patients and families (48–50), the interviews showed no examples of episodes involving direct attacks toward LGBTQ+ healthcare workers. Discrimination reported in this sense refers either to LGBTQ+ healthcare professionals witnessing other colleagues saying something about LGBTQ+ patients, or patients being indirectly abusive toward the LGBTQ+ community as a whole. In this sense, the choice of invisibility preferred by most HPs seem to function as a protective factor that prevents them from being recognizable by homo-transphobic patients and hence deactivate the potential conflicts that may arise. This seems to be the case in particular in a context where frustration against LGBTQ+ people and against the shortcomings of public healthcare are growing (11, 51).

### Between reluctance and resistance

The research shows that LGBTQ+ healthcare professionals navigate their identity at work oscillating between reluctance and resistance. On one side, most participants show some degree of reluctance in acknowledging that their visibility in the workplace could be beneficial for other LGBTQ+ people accessing healthcare, in particular patients. This role as visible ambassadors of equality and diversity in the healthcare system is not discussed nor contemplated, as HPs seem to focus primarily on the personal struggles of coming out and interprofessional relationships. The dilemmas around visibility (28) and the management of the LGBTQ+ identity seem to remain confined to an intimate sphere that is not shared at a collective level. Even when they know other LGBTQ+ colleagues, participants do not share conversations about discrimination or difficulties connected to their identities. These data may partially provide an explanation for the absence of formal organizations that reunite LGBTQ+ healthcare professionals in Portugal. This is a relevant absence considering that in other countries organizations of this kind have been active for years, as in the case of “GLADD—The Association of LGBTQ+ Doctors and Dentists” in the United Kingdom or “Roze in Wit” in the Netherlands. The reluctance to manage their LGBTQ+ identity in its collective dimension reflects also in the scarce use of complaint mechanisms or formal legal actions when HPs are victims of discrimination. The interviews show that HPs resort to these instruments only when the situations suffered are unequivocal, repeated, and severe: in all other situations, HPs tend to avoid direct confrontation, either ignoring the discrimination or suffering indirect consequences, for example on their mental health.

However, the reluctance to visible action is only one side of results emerging from the study: on another side, HPs show the ability to engage in a variety of micro-practices of resistance to the pressures of heteronormativity and homo-transphobia. If on a superficial and visible level they are mostly passive witnesses, on a daily and impalpable level they constantly operate choices to care for their wellbeing and protect their integrity. For example, they may silently avoid confrontation with a homo-transphobic colleague while at the same time strengthening emotional bonds with a few others that will provide comfort and protection. Or, they may provide additional attention and empathy when caring for an LGBTQ+ patient without making it visible or blatant. Moreover, HPs generally acknowledge that their visibility practices changed in time and, thus, may still change in the future: their negotiation of visibility in the workplace is a process in the making that does not ever end (8, 23, 52). These micro-practices of resistance may not do a significant, measurable difference on a large scale: however, they are signals high levels of awareness regarding the complexity of being LGBTQ+ as healthcare professionals.

The analysis overall shows that, beside the macro-level of interprofessional relationships and hierarchies in healthcare, negotiation of well-being, visibility, and care is done at a micro-level, in a smaller, less visible, less tangible terrain: in the quest to promote more inclusive and accessible healthcare spaces for LGBTQ+ people, this elusive element needs to be considered as crucial, with all the challenges it poses to research and policy-making.

### Limitations and future directions

As described in the methods section, this analysis is based exclusively on the qualitative data gathered in the context of a larger study involving also quantitative data. While the experiences discussed in the qualitative interviews show a richness and a depth that quantitative data cannot completely grasp., they will also acquire more significant once they are contextualized in a larger mixed-method analysis. Moreover, the sample that participated in the qualitative study is not representative of the whole diversity existing within the healthcare professions: in particular, the absence of participants from technical professions and assistant, as well as participants from the older cohorts of age (55+) has to be acknowledged as a limitation. Finally, the study reflects data gathered in Portugal, a country characterized by the compresence of a strong public healthcare system and a definite framework of protection of LGBTQ+ rights. Data should be understood as a reflection of this contextual frame, and may not be easily comparable with other studies, for example those involving HPs in the private sector.

Future directions in the study of LGBTQ+ healthcare professionals should encompass the influence of contemporary phenomenon occurring in several countries of the Global north, such as the increasing influence of distrust and science-denial in healthcare (32); the impact of far-right movements in the application of DEI programs in healthcare professions (51); and the role of organized mobilization around diversity in healthcare in changing the training for healthcare professionals (53).

## Data Availability

The raw data supporting the conclusions of this article will be made available by the authors, without undue reservation.
